# Human-based complex *in vitro* models: their promise and potential for rare disease therapeutics

**DOI:** 10.3389/fcell.2025.1526306

**Published:** 2025-01-27

**Authors:** Surat Parvatam, Francesca Pistollato, Lindsay J. Marshall, Fabia Furtmann, Devashree Jahagirdar, Mohua Chakraborty Choudhury, Sujata Mohanty, Harshita Mittal, Saveetha Meganathan, Rakesh Mishra

**Affiliations:** ^1^ Department of Research and Toxicology, Humane Society International/India, Hyderabad, India; ^2^ Department of Research and Toxicology, Humane Society International/Europe, Brussels, Belgium; ^3^ Animal Research Issues, The Humane Society of the United States, Washington DC, DC, United States; ^4^ Department of Chemical Engineering, Indian Institute of Technology (IIT), Mumbai, Maharashtra, India; ^5^ DST Centre for Policy Research, Indian Institute of Science, Bangalore, India; ^6^ Stem Cell Facility (DBT-Centre of Excellence for Stem Cell Research), All India Institute of Medical Sciences, New Delhi, India; ^7^ Community Engagement and Policy Stewardship, Tata Institute for Genetics and Society, Bangalore, India; ^8^ Tata Institute for Genetics and Society, Bangalore, India

**Keywords:** rare diseases, organoids, iPSCs, CIVM, cystic fibrosis, organ-on-chip, drug development

## Abstract

Rare diseases affect a small percentage of an individual country’s population; however, with over 7,000 in total, rare diseases represent a significant disease burden impacting up to 10% of the world’s population. Despite this, there are no approved treatments for almost 95% of rare diseases, and the existing treatments are cost-intensive for the patients. More than 70% of rare diseases are genetic in nature, with patient-specific mutations. This calls for the need to have personalised and patient-specific preclinical models that can lead to effective, speedy, and affordable therapeutic options. Complex *in vitro* models (CIVMs), including those using induced pluripotent stem cells (iPSCs), organoids, and organs-on-chips are emerging as powerful human-based pre-clinical systems with the capacity to provide efficacy data enabling drugs to move into clinical trials. In this narrative review, we discuss how CIVMs are providing insights into biomedical research on rare diseases. We also discuss how these systems are being used in clinical trials to develop efficacy models for rare diseases. Finally, we propose recommendations on how human relevant CIVMs could be leveraged to increase translatability of basic, applied and nonclinical research outcomes in the field of rare disease therapeutics in developed as well as middle-and low-income countries.

## 1 Introduction

Rare diseases are defined as diseases that affect a small percentage of a country’s population. The International Classification of Diseases (ICD), the legally mandated international health data standard by World Health Organization (WHO), includes 5,500 rare diseases. Collectively, these affect 6%–8% of the population, up to or 560 million people globally. In addition, 50% of the rare diseases affect children and can be severely debilitating and disabling, and 30% of those children die before 5 years of age. So, the impact of the rare diseases cannot be underestimated (Endocrinology, 2019).

While WHO defines a rare disease with a prevalence of 1 or less per 1,000 population, this definition of rare disease can vary based on the country (Richter et al., 2015). In the US, a rare disease is defined as one that affects 200,000 people or fewer, and more than 25 million Americans are affected by around 7,000 rare diseases. In Europe, rare diseases are defined as affecting no more than 1 in 2,000 people. There are more than 6,000 different rare diseases in the EU that are known to affect up to 36 million citizens. About 9% of Southeast Asia’s population is living with a rare disease (Shafie et al., 2016)—the higher rates of rare genetic disorders in these regions is often attributed to traditional marriage practices of endogamy and consanguinity (Angural et al., 2020; Wall et al., 2023). Under the New Drugs and Clinical Trial Rules (2019) in India, a rare disease is defined as a condition that affects not more than 500,000 persons and currently, about 72–96 million people in India live with a rare disease.

In addition, there are no approved treatments for 95% of rare diseases (The Lancet Global Health, 2024). In India, around 450 rare diseases have been identified but the therapeutics approved by the Drugs Controller General of India (DCGI) are available only for about 12–15 of them (Bureau, 2023; eClinicalMedicine, 2023).

Historically, there has been less initiative from pharmaceutical companies to take a leap into rare disease therapeutics, as the return on investment may be limited due to the low market share (Yates and Hinkel, 2022; Yoo, 2023). The US attempted to address this with the Orphan Drug Act, offering incentives for companies developing drugs for rare disease to make this activity more economically viable. This had some impact but there is still a pressing need to develop effective and affordable therapeutics for those ∼7,000 rare diseases that do not have approved therapies (Miller et al., 2021). However, there are several challenges associated with rare disease therapeutics, with animal models either unavailable, or often failing as preclinical test systems and thus providing limited insights regarding their biological mechanisms. For example, a gene therapy tested in the dog model for Myotubular myopathy, a rare muscular disease which affects 1 in 50,000 male newborns worldwide, showed promising results and prevented muscle pathology. However, the treatment led to severe liver problems in the patients, providing just one example that animal models often provide poor insights into disease mechanisms of rare diseases (May, 2023). One recent analysis indicated that incorporation of CIVMs into the preclinical workflow could increase productivity, generating an additional $3 billion annually for the pharmaceutical industry (Ewart et al., 2022). This could help to reduce treatment costs and increase access to rare disease treatments, especially for low and middle-income countries (Srivastava et al., 2024).

The use of genetically engineered animal models has become one of the main strategies for developing rare disease therapeutics; however, knock-out or genetically edited animals often do not exhibit key clinical characteristics observed in rare diseases (Nolan et al., 2021; Yang, 2023). For example, knock-in and knockout mouse models of age-related macular degeneration (AMD) and other allied macular dystrophies (MDs) typically lack the phenotypic manifestations seen in human patients (Nolan et al., 2021). While most macular dystrophies are autosomal dominant in humans, double dominant allele editing in mice has been necessary to even partially phenocopy the corresponding human pathology. Similarly, mutations in the gene *LRRK2* account for 5%–6% of patients with familial Parkinsons Disease (PD) and 1%–3% of sporadic cases, but most *LRRK2* mutant transgenic mouse models showed minimal or no neurodegeneration (Xu et al., 2012). In addition, even within a single rare disease, many subtypes can exist. For example, there are more than 2,000 known *CFTR* (cystic fibrosis transmembrane conductance regulator) gene variants with variable (asymptomatic to severe) clinical manifestations (Castellani et al., 2008). Thus, creating large numbers of animal models with different sets of genetic mutations for a particular rare disease can become very challenging, costly and time-consuming. Another significant limitation of genetically engineered model organisms is that, apart from the edits in the gene loci of interest, the rest of the genetic background is representative of the model organism, and not humans. Several studies have highlighted how genetic backgrounds can significantly impact the phenotype or the clinical manifestation of a disease (Research, N. R. C. I. C. of the I. for L. A and Richter, 1999; Olguín et al., 2022).

Conducting clinical trials for rare diseases also presents challenges due to the small patient pool and the reluctance of patients to participate in a clinical trial without the opportunity of receiving an investigational product (Mellerio, 2022). These treatments are often lifelong with the drug dose and cost increasing with age and weight (Yang, 2023), which places immense strain on the resources of the patients.

These complexities point towards the urgent need to develop innovative approaches that provide personalised and human-relevant models for rare diseases. Advances in cell and molecular biology in the last decade have led to the development of human-cell based complex *in vitro* models (CIVMs), including those that employ patient-derived induced pluripotent stem cells (iPSCs), spheroids, organoids, or organ-on-chip (microphysiological systems or MPS) technologies. These systems can help to mimic the human disease mechanisms at a cellular and tissue level, allowing mechanistic assessment of the drug response and toxicity in the context of the genetic background of a patient. With 71.9% of rare diseases being genetic in nature (Nguengang Wakap et al., 2020), these CIVM systems can be leveraged to more accurately model the disease phenotype and develop patient-specific therapeutic strategies.

While certain rare diseases require relatively low-cost, albeit lifelong interventions, such as specific dietary formulation, enzyme replacement therapy, hormonal therapy (Tambuyzer et al., 2020), for the purposes of this review, we focused on specific rare diseases for which optimal interventions, based on cost and/or positive long-term outcomes, are currently lacking. In addition, the rare diseases covered in this review also are of global concern and prevalence. We provide examples of how human-relevant, complex *in vitro* models (CIVM), such as patient-derived iPSCs, organoids and MPS have proven effective in enabling biomedical research and drug development for these rare diseases. We take a brief look at where clinical trials for this group of rare diseases are employing CIVMs and summarise how they are currently being used. Finally, we provide recommendations to accelerate drug development for rare diseases using these emerging technologies globally.

## 2 Complex *in vitro* model-based tools to study rare disease patho-mechanisms

In the following sections, we present examples of studies describing how CIVMs, such as human iPSCs and MPS, have been instrumental in modeling rare diseases, focusing on lysosomal storage disorders, cystic fibrosis, Duchenne muscular dystrophy, spinal muscular atrophy, Ehlers-Danlos syndrome, multiple sclerosis, and inherited retinal dystrophies. We explore how these human cell-based models have contributed to advancing our understanding of the mechanisms underlying the pathophysiology of these conditions.

### 2.1 Lysosomal storage disorders

Lysosomal storage disorders (LSD) consist of more than 70 inherited metabolic disorders due to defects in the genes that encode for lysosomal proteins. Within the lysosome, hydrolytic enzymes degrade macromolecules, such as lipids, carbohydrates, proteins etc., into their terminal components, including fatty acid, monosaccharides, amino acids. However, deficiency in a single hydrolase can create an inability to degrade these macromolecules, leading to lysosomal storage diseases (Ferreira and Gahl, 2024). Based on the storage material that is affected, LSD can be further sub-divided into sphingolipidoses, mucopolysaccharidoses, glycoproteinoses, lipid storage disease, and glycogen storage disease (Sun, 2018). Sphingolipidoses affect the enzymes required for lipid catabolism; in the mucopolysaccharidoses, glycosaminoglycans are not degraded effectively so they accumulate in cells and tissues; and glycoproteinoses refer to those conditions where undegraded oligosaccharides build up in the lysosome. LSD can also be sub-classified, based on the age of onset of the disease, as infantile, juvenile, or adult-onset forms. The worldwide incidence of LSD is 1 in 5,000–7,500 births (Platt et al., 2018); however, in certain populations, this incidence may vary. For example, the Ashkenazi Jewish population has a high frequency of 1 in 855 births for Gaucher disease (Staretz-Chacham et al., 2009).

#### 2.1.1 Gaucher disease (GD)

Gaucher disease (GD**)** is an LSD caused by mutations in the b-glucocerebrosidase (*GBA1*) gene, located on chromosome 1, leading to deficiency of the lysosomal enzyme, glucocerebrosidase. This enzyme hydrolyses glucosylceramide into ceramide and glucose, and its deficiency causes an accumulation of glucosylceramide in macrophages (Stirnemann et al., 2017). While the phenotypes associated with this disease are variable, three clinical forms have been identified. The most common variant, type 1, is characterised by effects on the viscera, whereas types 2 and 3 are associated with neurological impairment. Researchers have been able to generate human induced pluripotent stem cells (hiPSC) from patients with type 1, type 2, and type 3 GD that effectively recapitulate the pathologic hallmarks of the disease (Panicker et al., 2012). GD patient-derived iPSCs were used to investigate the mechanism underlying *GBA1*-associated neurodegeneration. These cells demonstrated widespread lysosomal depletion and a blockage in autophagic flux, resulting from impaired clearance of autophagosomes by lysosomes (Awad et al., 2015). In addition, mutations in *GBA1* were associated with downregulated expression of transcription factor EB (TFEB), the master regulator of lysosomal genes, and its target genes. The study concluded that lysosomal defects as a consequence of glucocerebrosidase deficiency are potentially major determinants of neurodegeneration in GD. Using hiPSC as a model system, several studies have shown that autophagy and inflammation associated with LSD have significant implications in developing therapeutics for LSD (Son et al., 2015; Luciani et al., 2020). Studies also showed that patients with Type I GD are at an increased risk of Parkinson’s diseases (PD) (Neudorfer et al., 1996); however, the mechanism is not clear. iPSC-derived neurons from GD and PD individuals carrying *GBA1* mutations exhibited increased levels of α-synuclein (Schöndorf et al., 2014). Additionally, they showed that complex changes in the autophagic/lysosomal system and calcium homeostasis could help to explain the heightened vulnerability of midbrain dopaminergic neurons to PD.

#### 2.1.2 Sandhoff disease

Sandhoff disease is an extremely rare lysosomal storage disorder characterised by the accumulation of GM2 gangliosides in the brain. This occurs due to a deficiency in the activity of hexosaminidase A (HEXA) and hexosaminidase B (HEXB) (Marques and Saftig, 2019). Accumulation of GM2 gangliosides leads to neurodegeneration and early childhood death in Sandhoff disease. To investigate the underlying mechanisms, researchers created an early neurodevelopmental model by generating brain organoids from iPSCs derived from a Sandhoff patient’s fibroblasts (Allende et al., 2018). Sandhoff disease brain organoids exhibited GM2 ganglioside accumulation and impaired neuronal differentiation, in contrast to the isogenic controls. This indicated that iPSC-derived brain organoids could recapitulate the pathological signatures of the Sandhoff disease.

#### 2.1.3 Fabry disease

Fabry disease is a rare X-linked lysosomal storage disorder caused by mutations in the *GLA* gene which encodes the α-GalA enzyme (Waldek and Feriozzi, 2014). The deficiency in α-GalA leads to the accumulation of globotriaosylceramide (Gb3) and related neutral glycosphingolipids inside lysosomes, disrupting cellular morphology and function (Waldek and Feriozzi, 2014; Luciani et al., 2020). Researchers created *GLA*-knockout (KO) human iPSCs and used these to develop kidney organoids that recapitulated Fabry disease phenotypes. These organoids exhibited deformed podocytes and tubular cells, along with accumulated Gb3 (Kim et al., 2021). In addition, *GLA*-KO kidney organoids displayed increased oxidative stress and apoptosis. The authors demonstrated that enzyme replacement treatment (ERT) with recombinant human α-Gal A could reduce Gb3 accumulation and alleviate oxidative stress, leading to the rescue of the *GLA*-KO kidney organoids deformed structure in Kim et al. (2021). This study highlighted kidney organoids as a valuable tool for exploring therapeutic options for Fabry disease. Clinically, ERT has been shown to significantly reduce microvascular deposits, alleviate pain and improve the quality of life in Fabry disease patients. However, further research is needed to determine the optimal form, dosage, frequency, and long-term effects on morbidity and mortality (El Dib et al., 2016).

### 2.2 Cystic fibrosis

Cystic fibrosis (CF) is an autosomal recessive disease that is caused by mutations in the Cystic Fibrosis Transmembrane Conductance Regulator (*CFTR*) gene. The CFTR is a chloride and bicarbonate ion channel that regulates transport through the apical membrane of most epithelial cells. Around 2000 CFTR gene mutations, have been identified, of which, only around 200 have been characterised (De Boeck and Amaral, 2016).

*CFTR* mutations are categorised into six classes and can lead to a broad range of phenotypes (Veit et al., 2016). CF affects multiple organs, including the lungs, pancreas, liver, intestines, and the reproductive tract (Dumas et al., 2021). However, many of these pathologies are not mimicked in animal models. For example, lung disease in people with CF is associated with chronic infection, inflammation, airway remodeling, and mucus obstruction; whereas, CF mice have normal lung function (Semaniakou et al., 2019).

The dysregulation of chloride ion secretion due to mutations in *CFTR* leads to an imbalance in ion and water transport that leads to the production of dehydrated mucus, inhibition of the mucociliary escalator, and enhanced neutrophilic inflammation (Stoltz et al., 2015; Martin et al., 2018). This predisposes the lungs to recurring cycles of infection, leading to cell damage and eventually, a decline in lung function (Szczesniak et al., 2023). The over-production of proinflammatory cytokines, such as IL-8 (Tabary et al., 1998) in the airways drives an exaggerated inflammatory cycle, attracting large numbers of polymorphonuclear neutrophils to the airways. The release of extracellular mediators by the neutrophils further exacerbates inflammatory cell recruitment and contributes to cell damage in the airways (Forrest et al., 2018). One research group created healthy and CF airway-on-chip models lined with primary human bronchial or bronchiolar epithelial cells and human lung microvascular endothelial cells from healthy subjects or people with CF (Plebani et al., 2022). Both the healthy and CF airway chips demonstrated functionality with highly differentiated epithelium containing ciliated, basal, club, and goblet cells. Importantly, the CF chip recapitulated key disease features, including a higher percentage of ciliated cells, increased ciliary beat frequency, more mucus accumulation, elevated baseline inflammation, and enhanced neutrophil recruitment through increased IL-8 secretion. These findings highlight the potential of using this approach as a preclinical tool for understanding the pathophysiology of CF and drug screening.

Cell lines generated from iPSCs derived from CF patients with specific mutations, including F508del (Kondrateva et al., 2021), Asn1303Lys (Merkert et al., 2020), and S308X (Khor et al., 2022) also offer valuable resources for disease modeling and research on the pharmacological response to CFTR modulators (Cheng et al., 2023).

CF is a multi-organ condition, and CIVMs can also help in modelling the pathologies in different organs. For example, the development of a pancreas on a chip (*39*), where patient-derived pancreatic ductal epithelial cells (PDECs) are co-cultured with islet cells, was used to reveal a CFTR-dependent reduction in insulin secretion (Shik Mun et al., 2019).

CFTR is also expressed in vascular endothelial cells, and mutations in *CFTR* can compromise endothelial function (Totani et al., 2017). A 2021 meta-analysis investigated whether endothelial-to-mesenchymal transition (EMT) occurs in CF endothelial cells, given that the mesenchymal transition of epithelial cells (EMT) has been linked to CF disease progression (Treps et al., 2021). Researchers performed a transcriptomic meta-analysis comparing CFTR-impaired and CF-derived endothelial cells with CF epithelial cells, where EMT is already well-established. The analysis revealed that CFTR-impaired endothelial cells exhibited a limited EMT signature, with no change in the expression of the transcription factor and mesenchymal inducer Twist1. However, treatment with CFTR modulators reduced the expression of mesenchymal markers in CF patient-derived endothelial cells, suggesting potential therapeutic benefits of CFTR modifiers beyond correcting defective CFTR-mediated ion transport (Treps et al., 2021).

Patient-derived intestinal organoids can be used to stratify patients for CFTR modifier therapies (Dekkers et al., 2013). These organoids retain the epigenetic signatures of individuals, which is important for a genetic disease like CF, where the impact of *CFTR* mutations and disease severity may vary widely. A recent study used patient-derived organoids from over seventy different donors to screen FDA-approved drugs for potential CFTR modifier activity. The study concluded that this platform could be used to identify responsive patients and effective treatments (de Poel et al., 2023). In 2017, the US FDA extended the approval of the Vertex Pharmaceuticals’ cystic fibrosis (CF) drug ivacaftor (CFTR modifier) to a broader range of mutations, based solely on *in vitro* data (Kingwell, 2017). This development was highly significant, as it accelerated the access to these breakthrough drugs to a larger population of CF patients. Importantly, it also paved the way for broader adoption of CIVMs in drug screening, particularly in cases like CF, where there is mechanistic understanding of the rare disease and a clinically relevant functional readout.

### 2.3 Duchenne muscular dystrophy

Duchenne muscular dystrophy (DMD) is a hereditary neuromuscular disease and one of the most common forms of muscular dystrophy. Global statistics show that approximately 1 in 5,000 male births are diagnosed with DMD (Mendell and Lloyd-Puryear, 2013). As stated earlier, most rare diseases are genetic and in the case of DMD, a recessive mutation in the dystrophin gene located on chromosome Xp21 leads to progressive muscle fiber degeneration and weakness. Dystrophin is the largest human gene comprising roughly 0.1% of the entire genome (Koenig et al., 1987). Deletion of one or more exons accounts for 60%–70% of DMD cases and 80%–85% of Becker muscular dystrophy (BMD) cases (Koenig et al., 1989). Point mutations and exon duplications result in 26% and 15% of DMD cases, respectively. Dystrophin is predominantly expressed in skeletal and cardiac muscles, as well as in the brain and retina (Gao and McNally, 2015; Wells, 2019; Kaplan and Morgan, 2022). It plays a crucial role in strengthening muscle fibers and protecting them from injury during contraction and relaxation.

DMD is a progressive disease with a poor prognosis, and effective therapeutic interventions remain scarce. Despite significant advances in medical research and technology, no reliable, cost-effective treatment is currently available for DMD. Elevidys, a gene therapy approved by the FDA in 2023, costs $3.2 million per dose, making it economically unfeasible. Moreover, it was recently reported that Elevidys, which received FDA accelerated approval, failed to show statistically significant improvement in motor function compared to placebo (Spuler et al., 2024). At present, most treatment options focus on alleviating symptoms and temporarily slowing disease progression.

A 3D skeletal muscle model constructed from iPSC derived from healthy donors and DMD patients was able to accurately replicate the characteristics of DMD (Maffioletti et al., 2018). This model integrated fully human, iPSC-derived, complex, multilineage muscle constructs containing key isogenic cellular constituents of skeletal muscle, such as vascular endothelial cells, pericytes, and motor neurons. By differentiating iPSCs to the late myogenic stage, the model successfully recapitulated the classical DMD phenotype. Treatment with prednisolone, the standard of care for DMD, restored the defects in contractile force generation by skeletal muscle and reversed the abnormal branching observed in DMD myofibers (Al Tanoury et al., 2021).

Many lines of research have focused on restoring dystrophin in muscle membranes for treating DMD. In 2016, a study developed microengineered DMD model of skeletal muscle to assess the ability of human mesoangioblasts, compared to human myoblasts, in restoring dystrophin levels and distribution along the myotube when cocultured with myoblasts derived from DMD patients (Serena et al., 2016). The results showed that mesangioblasts could restore dystrophin expression on DMD myotubes, offering an *in vitro* platform that could be used to assess new approaches that can enhance dystrophin accumulation and distribution.

Dilated cardiomyopathy is one of the leading causes of death in DMD, but its pathogenesis remains poorly understood. A patient-derived cardiac organoid model that displayed cardiomyopathy and disease progression phenotypes during long-term culture has been shown to be a valuable tool to study DMD-related cardiomyopathies (Marini et al., 2022; Przymuszała et al., 2024).

Shahriyari et al. reported a protocol for transgene-free and serum-free engineered skeletal muscle (ESM) that closely mimicked the developmental trajectory for muscle formation, responded to developmentally relevant cues, and recapitulated the contractile deficit of DMD (Shahriyari et al., 2022).

These studies highlight the methods and tools that are being developed to investigate specific endpoints and pathologies associated with DMD, such as cardiomyopathy, dystrophin deficiency, and diminished contractile performance. Thus, CIVMs, or a combination of CIVMs, could be used to examine these, and other pathologies related to rare diseases more broadly.

### 2.4 Spinal muscular atrophy

Spinal Muscular Atrophy (SMA) is a hereditary, neuromuscular disease that characterised by the degeneration of alpha motor neurons in the anterior horn of the spinal cord, leading to progressive muscle weakness and is ultimately fatal (Aasdev et al., 2024). SMA is classified into five subtypes based on the age of onset and the degree of motor function that is affected (Prior et al., 1993). Type 0 SMA, the most severe form, presents prenatally and is fatal in the first 6 months of life. Affected infants require respiratory support and exhibit severe muscle weakness and hypotonia. Type 1(Werdnig-Hoffmann disease) is acute and severe, accounting for up to 80% of SMA cases. Its onset occurs before 6 months of age, marked by a distinct bell-shaped upper body, and average survival is around 2 years. Type 2 (Dubwitz disease) typically manifests between 7–18 months of age and survival to early adulthood is possible, with protective care. Type 3 (Kugelberg-Welander disease) individuals usually have a normal lifespan with supportive care, though motor function declines slowly, starting in early adulthood. Type 4 is an adult-onset form affecting fewer than 5% of patients, with motor function impairment occurring after the age of 30 (Aasdev et al., 2024).

SMA is an autosomal recessive disorder most commonly caused by homozygous deletion or deletion and mutation of the alleles of the survival motor neuron 1 (*SMN1*) gene located on chromosome 5q13 (Lefebvre et al., 1995). While deletion or mutation of *SMN1* results in SMN1 protein deficiency, the *SMN2* gene produces a relatively small amount of functional SMN protein and *SMN2* copy numbers modulate the severity of the disease (Prior et al., 2009). As of 2017, the incidence of SMA is approximately 10 in 100,000 live births (Verhaart et al., 2017).

Current *in vitro* models for SMA include patient-derived spinal organoids developed from iPSCs. Comparative analysis of iPSCs from SMA and healthy individuals that were differentiated into motor neurons revealed upregulation of several cyclins and cyclin-dependent kinases (CDKs) in iPSCs from SMA patients (Hor et al., 2018). Furthermore, treatment with small molecule inhibitors of CDKs was shown to prevent motor neuron degeneration in SMA, suggesting that small molecules could serve as potential therapeutic strategies for SMA.

Another study used isogenic patient-derived induced pluripotent stem cell (iPSC) model and a spinal cord organoid (SCO) system to show that early neurodevelopmental defects may underlie later spinal motor neuron (MN) loss and muscle wasting observed during SMA, further indicating that postnatal MN-increasing interventions may not rescue the SMA pathology (Grass et al., 2024). These studies demonstrate how relevant CIVMs can provide immense insights into the pathology and treatment strategy for SMA.

### 2.5 Ehlers-danlos syndrome

Ehlers-Danlos syndrome (EDS) comprises a group of rare inherited connective tissue disorders, characterised by hyperextensible skin, joint hypermobility, and tissue fragility (Hakim, 1993). Vascular EDS (vEDS), a subtype of this condition, is caused by mutations in the *COL3A1* gene (Ruscitti et al., 2021), which encodes type III collagen, an essential component of blood vessel walls. vEDS can be life-threatening, as patients are at risk of severe internal bleeding due to arterial ruptures. Recent studies have highlighted the potential of patient-derived iPSC models to investigate the underlying pathophysiology of vEDS. For instance, peripheral blood mononuclear cells (PBMCs) from a patient with a heterozygous nonsense mutation, c.430C > T (p.Q105*), in the *COL3A1* gene, were reprogrammed into iPSCs (Höpperger et al., 2024). Similarly, iPSC lines were generated from two vEDS patients with a c.226A > G, p. Asn76Asp missense mutation in the *COL3A1* gene (Manhas et al., 2024).

Induced PSCs have been also generated from skin fibroblasts of three patients with musculo-contractual EDS (mcEDS), which is caused by mutations in the carbohydrate sulfotransferase 14 gene (*CHST14*). A patient-specific iPSC-based human osteogenesis model was developed to study the phenotype and pathophysiology of mcEDS. The patient-derived iPSCs exhibited significantly downregulated osteogenic-specific gene expression and reduced calcium deposition compared to wild-type iPSCs, underscoring the role of *CHST14* dysfunction in the development of skeletal deformities (Yue et al., 2023).

### 2.6 Multiple sclerosis

Multiple Sclerosis (MS) is a chronic, immune-mediated inflammatory and neurodegenerative disease that primarily affects the central nervous system (CNS). It is characterised by demyelination—the destruction of the protective sheath surrounding nerve fibers—along with inflammation and progressive neurodegeneration, leading to a range of neurological symptoms. MS manifests in various forms, with the two most common being relapsing-remitting MS (RRMS), where patients experience episodes of symptom recovery; and primary progressive MS (PPMS), which is marked by a steady worsening of symptoms without clear relapses. Research has emphasised the complex interplay between genetic predisposition, environmental factors, and immune system dysregulation in driving the pathology of MS (Waubant et al., 2019).

A 2023 study used iPSC-derived cerebral organoids to explore cellular mechanisms in PPMS patients (Daviaud et al., 2023). A key finding was the reduction in both SOX2+, a transcription factor crucial for maintaining stem cell pluripotency, and the proliferation marker Ki67, suggesting a compromised regenerative capacity in PPMS. Additionally, the study reported premature neuronal differentiation at the expense of oligodendrocyte formation, remyelination failure, and dysregulated expression of the cell cycle inhibitor p21, exacerbating stem cell dysfunction. The study emphasised the influence of genetic background on stem cell behavior, underscoring the importance of personalised medicine approaches for understanding the pathology and treatment of individuals with MS.

Another study provided insights into astrocyte dysfunction in MS pathology by examining metabolic changes in astrocytes derived from iPSCs of MS patients (Ghirotto et al., 2022). Metabolic alterations included increased oxidative stress markers, such as elevated superoxide production, suggesting that astrocytes in MS contribute to neuroinflammation and neurodegeneration. The study also reported dysfunction in mitochondrial and sphingolipid metabolism, indicating impaired energy metabolism and a proinflammatory astrocyte phenotype. These findings inform potential therapeutic targets through modulation of metabolic pathways and oxidative stress responses to mitigate astrocyte-related neuronal damage in MS. Moreover, astrocytes were found to have a dual function in MS. Reactive astrocytes derived from iPSCs from benign and progressive MS patients secreted neurotrophic factors and exhibited neuroprotective properties when co-cultured with neurons exposed to inflammatory cytokines, such as TNF-α and IL-1β. The ability of iPSC-derived reactive astrocytes to protect neurons in inflammatory conditions opens new avenues for therapeutic strategies in MS.

Another study successfully generated iPSCs from patients with RRMS and PPMS, confirming their ability to differentiate into key cell types, including neurons and oligodendrocytes, thereby establishing a reliable model for MS research (Mutukula et al., 2021). Functional assays demonstrated that MS-derived oligodendrocytes exhibited altered properties compared to controls, which is consistent with the pathology observed in MS. This iPSC-based platform also holds potential for screening remyelinating drugs, offering a valuable tool for advancing therapeutic strategies for MS (Fortune et al., 2022).

### 2.7 Inherited retinal dystrophies

Inherited retinal dystrophies (IRDs) are a group of degenerative disorders of the retina characterised by clinical and genetic heterogeneity. Common phenotypic features include color or night blindness, peripheral vision impairments, and eventual progression to complete blindness. For studying IRD, methods have been established to guide the differentiation of iPSCs into authentic retinal cells using both two-dimensional adherent cultures and three-dimensional organoid systems. Innovative approaches to studying IRDs, such as choroideremia, cone-rod dystrophy, retinitis pigmentosa, and other diseases, have provided crucial insights into the molecular and cellular mechanisms underlying these currently untreatable conditions (Faynus and Clegg, 2022; Liang et al., 2023).

Recently, iPSCs were generated from a patient with early-onset pattern retinal dystrophy attributed to a heterozygous mutation in the *orthodenticle homeobox 2* (*OTX2*) gene (Zhang et al., 2024). *OTX2* gene variants are linked to a wide range of ocular disorders, including IRDs (Gregory et al., 2021). These iPSCs were successfully differentiated into the three primary germ layers, as well as retinal organoids and retinal pigment epithelial cells (Zhang et al., 2024).

Mutations in the *eyes shut homolog* (*EYS*) gene are also associated with IRDs. Retinal organoids generated from iPSCs derived from *EYS*-associated IRD patients were characterised by the absence of EYS and G protein-coupled receptor kinase 7 (a key protein involved in managing phototoxicity from the outer segment of photoreceptor cells) (Otsuka et al., 2024). Additionally, photoreceptor cells in these organoids were more susceptible to light-induced damage, suggesting that strategies directed at reducing phototoxicity could be promising for *EYS*-associated IRDs.

Choroideremia (CHM) is a rare X-linked retinal disorder caused by mutations in the *CHM* gene, leading to progressive vision loss due to degeneration of the choroid, retinal pigment epithelium (RPE), and retina. The RPE, through its support and maintenance of the photoreceptors, is vital in normal retinal health, thus RPE degeneration plays a key role in disease progression. Commonly used models, like the human RPE cell line ARPE-19 do not accurately reproduce the features of mature RPE cells such as melanin accumulation and are inadequate for studying CHM. However, human iPSCs can be differentiated into RPE cells that more closely resemble native RPE. A 2024 study describes the creation of two isogenic hiPSC lines using CRISPR/Cas9 (Fonseca et al., 2024) that exhibited critical features of native RPE, such as tight junction markers, phagocytosis of photoreceptor segments, pigmentation, and a postmitotic state. Furthermore, these cells were able to form retinal organoids. This work represents the first development of isogenic hiPSC lines for CHM, providing a valuable tool for understanding the mechanisms driving RPE degeneration in the disease and opening possibilities for future therapeutic exploration.

Retinitis pigmentosa (RP) is a heterogenous group of IRDs that leads to progressive vision loss, affecting approximately 1 in 3,000–4,000 individuals worldwide. Patient-derived iPSCs have been increasingly used to model RP, providing valuable insights into its pathogenesis (Leong and Sowden, 2023; Lin et al., 2024). Notably, approximately 25% of autosomal dominant RP cases are caused by mutations in the rhodopsin (*RHO*) gene. A study using RP patient-derived retinal organoids, found that these organoids exhibited photoreceptor dysgenesis, increased *RHO* mRNA expression, and mislocalisation of the rhodopsin protein within rod photoreceptors (Kandoi et al., 2024) Additionally, treatment with the small molecule Photoregulin 3 partially corrected the mislocalisation of rhodopsin by targeting *NR2E3*, an *RHO* upstream regulator. These findings provide proof-of-principle for using patient-specific iPSC-derived retinal models to explore personalised medicine strategies, offering potential for targeted therapies for RP and other related IRDs.

## 3 Current usage of complex *in vitro* models in clinical trials for rare disease therapeutics

In 2023, Sanofi SA’s sutimlimab (SAR-445088; formerly BIVV-020), a humanised anti-C1s monoclonal antibody, was approved for conducting clinical trials for chronic inflammatory demyelinating polyneuropathy (CIDP), a rare neuromuscular disease, using efficacy data generated using an organ chip model (Rumsey et al., 2022). This was one of the first cases where an IND application was approved for conducting clinical trials based on efficacy data purely generated from CIVM.

To understand whether CIVMs are being used more widely in clinical trials, a search was performed on the clinical trials registries of the US (ClinicalTrial.gov) and Europe (clincialtrialregister.eu) to identify clinical trials that have used or are currently using CIVMs for rare diseases. The registries were searched using specific terms, including “*in vitro*” OR “organoid” OR “organ chip”, along with the name of each rare disease mentioned above. Hits were downloaded and further examined to ensure that CIVM were being used as part of the diagnostic or predictive elements. Searches were carried out between August 24 and 30 September 2024. It is relevant to highlight that this list is not exhaustive and is presented as an indicator of the trends of research rather than a definitive study of how CIVM are being applied for clinical research.

Table 1 shows the results of this preliminary analysis and indicates that there are several, ongoing clinical trials assessing the capacity of organoids to predict human drug responses in the context of rare diseases. There are currently 11 clinical trials for CF that are using CIVMs and are targeting the less common CF mutations, where there is greatest need for novel therapies. Around 15% of people with CF represent more than 2,000 different rare and mostly uncharacterised *CFTR* mutations. The European Union’s Horizon 2020 program has funded The Human Individualised Therapy of CF (HIT-CF), which aims to target the subset of patients carrying rare CF mutations (ClinicalTrials.gov ID: NCT06468527). This study consists of a two-step approach where the first step tests novel CFTR modulators and their combinations on organoids from over 500 European and Israeli people with CF. Based on the response in the organoids, the second part of the project will assess the therapeutic effect; preliminary completion data are expected for this trial in April 2025. Three other trials look at specific *CFTR* mutations, A455E (EU Clinical Trials Register ID: 2016-001585-29), R334W (ClinicalTrials.gov ID: NCT04254705), class III mutation (EU Clinical Trials Register ID: 2014-000817-30) and aim to assess the correlation between organoid-based outputs and clinical response.

**TABLE 1:** Use of CIVMs in clinical trials for rare diseases. The table provides results of a preliminary search (as of 10 October 2024) of the clinical trials registries of the US (www.clinicaltrials.gov) and EU (www.clinicaltrialsregister.eu/) for those trials intending to use *in vitro* methods for the rare diseases described.

Rare disease	ClinicalTrials.gov ID/EudraCT number	Name of the project	Start date/Status	Interventional/Observational	Usage of organoids/iPSCs in the clinical trials	Trial results (if completed)
CF	NCT06468527	Clinical Trial to Evaluate the Efficacy and Safety of Dirocaftor/ Posenacaftor/Nesolicaftor in Adults With CF (CHOICES)	Ongoing	Interventional: Dirocaftor/Posenacaftor/Nesolicaftor	(i) Performing a two-step approach, where in the first step, novel CFTR modulators/combinations were tested on organoids from over 500 European and Israeli CF patients with rare *CFTR* mutations to identify patients predicted to clinically benefit from these treatments. The second step will evaluate the predicted clinical effect in subjects identified by their organoid response	NA
CF	2015-001317-28 (Netherlands - Competent Authority Clinical Trial Type: EEA CTA)	A B2-agonist as a CFTR activator in CF	Prematurely Ended	Interventional: Salbutamol	(i) Assessing the correlation between B2-agonist induced CFTR function in organoids and long term clinical treatment effect on lung function and airway resistance) (ii) Examining the CFTR-stimulating potential of salbutamol concentration in the blood using organoids	No results available
CF	2016-001585-29 (United States)	A Phase 2, Randomised, Double-Blind, Placebo-Controlled, Crossover Study to Evaluate the Efficacy of Lumacaftor/Ivacaftor Combination Therapy in Subjects With Cystic Fibrosis Who Have an A455E-*CFTR* Mutation	Completed	Interventional: Lumacaftor/Ivacaftor	(i) Exploring the association between LUM/IVA-induced CFTR function in vitro organoid-based measurements and clinical response to LUM/IVA	No results available for organoid data
CF	2016-001619-19 (Netherlands - Competent Authority)	Genistein as an add-on treatment for CF?	Ongoing	Interventional: Genistein	(i) Assessing correlations between individual Ivacaftor genistein induced CFTR function *in vitro* (organoid-based measurements) and *in vivo* treatment affect	NA
CF	2014-000057-37 Netherlands - Competent Authority	A B2-agonist as a CFTR activator in CF	Completed	Interventional: Salbutamol	(i) Investigating the correlations between individual B2-agonist-induced CFTR function *in vitro* (using organoid-based measurements) and treatment effect *in vivo* (ii) Assessing if the dosage of B2-agonist used in the clinical study is sufficient to stimulate CFTR function, by testing the CFTR stimulating effect of patients’ blood samples *in vitro*, on autologous organoid	No results available
CF	2014-000817-30 Netherlands - Competent Authority	Comparing the effect of curcumin and genistein to treatment with Ivacaftor in CF patients with a class III mutation	Completed	Interventional: Curcumin and genistein	(i) Evaluating the correlations between individual curcumin + genistein and Ivacaftor induced CFTR function *in vitro* (organoid-based measurements) and the *in vivo* treatment effect (ii) Assessing if the dosage of curcumin + genistein and Ivacaftor used in the clinical study is sufficient to stimulate CFTR function using organoids	No results available for organoid data
CF	NCT03506061 (United States)	Trikafta in Cystic Fibrosis Patients	Completed	Interventional: Trikafta	(i) Establishing correlation between *in vitro* Trikafta responsiveness of iPSCs and *in vivo* benefit (FEV1), subsequently providing a new tool for utilizing iPSCs to identify patient populations most suitable for CF modulator therapy	No results available
CF	NCT04254705 (Universitaire Ziekenhuizen KU Leuven, The Netherland)	Organoid Study R334W	Withdrawn	Interventional: Tezacaftor + Ivacaftor	(i) Investigating response to CFTR modulators in organoids of 30 patients with CF and a R334W mutation, to allow further stratification for a future clinical trial	No results available
CF	NCT03390985 (Canada)	Canadian Observation Trial in CF Patients Undergoing Treatment With Ivacaftor (G551D)	Completed	Interventional: Ivacaftor	(i) Conducting functional *in vitro* test: Collected nasal brushes for CFTR to generate nasal cell cultures for the assessment of ivacaftor response and CFTR function	No results available
CF	NCT05100823 (University Hospital, Montpellier, France)	Validation of Therapeutic Efficacy Targeting the Splicing Variants in Cystic Fibrosis and CFTR Pathologies (ONB-CFTR)	Ongoing	Interventional: Oligonucleotide blockers (ONB)	(i) Assessing ONB (named ONB-CFTR) to be performed using an air-liquid interface model of airway epithelium, developed from nasal cells of patients, without or with a combination of existing CFTR modulators, depending on the patient’ genotype (ii) Building a local biobank of rectal organoids from CF patients	NA
CF	NCT03754088 (University Hospital, Montpellier, France)	*In Vitro* Model of the Cystic Fibrosis Bronchial Epithelium Via iPS Technology (PaCyFIC)	Completed	Observational	(i) Creating iPSC-derived bronchial epithelium from CF patients homozygous for the p.Phe508del *CFTR* mutation	No results available
MS	NCT06551649 (Rome, Italy)	Human Amniotic Mesenchymal Cell Secretome for Neurodegeneration and Neuroinflammation (CONTRASTOME)	Not yet recruiting	Interventional (no specific drugs or compounds are mentioned)	(i) Developing a three-dimensional cellular model of MS, including neurite length measurement, apoptosis, damaged myelin, number of inflamed microglial cells (ii) Evaluating the impact of human amniotic mesenchymal stromal cell secretome on above mentioned parameters of organoids	NA
DMD	NCT02413450 (Johns Hopkins University, United States)	Derivation of Human Induced Pluripotent Stem (iPS) Cells to Heritable Cardiac Arrhythmias	Enrolling by invitation	Observational	Generating iPSCs and iPSC-derived cardiomyocytes from blood or skin biopsies derived from patients and healthy controls to study pathogenesis and therapeutics	NA
SMA (and other neurological diseases)	NCT00874783 (Hadassah Medical Organization, Israel)	Development of iPS From Donated Somatic Cells of Patients With Neurological Diseases	Active, not recruiting	Observational	(i) Developing iPSCs from skin biopsies or the patient’s hair to model diseases and drug discovery (ii) assessing use of iPSCs for future transplantation therapy	NA
IRDs	NCT03853252 (University Hospital, Montpellier, France)	iPS Cells of Patients for Models of Retinal Dystrophies (RETIPS)	Recruiting	Interventional: gene therapy	Generating iPSC-derived retinal models obtained from healthy controls and patients to evaluate gene therapy efficiency for different IRDs	NA
IRDs	NCT01432847 (National Eye Institute (NEI), United States)	Cell Collection to Study Eye Diseases	Recruiting	Observational	Generating iPSC-derived RPE and/or neural retinal cells obtained from IRD patients and healthy volunteers to investigate molecular mechanisms involved in disease initiation and progression, and to perform high throughput drug screening	NA
IRDs	NCT02162953 (Mayo Clinic, United States)	Stem Cell Models of Best Disease and Other Retinal Degenerative Diseases	Completed	Observational	Generating iPSC-derived RPE from patients with Best disease or other IRDs to test therapeutic approaches	No results available
IRDs	NCT04658251 (University Hospital, Lille, France)	Study of New Mutations in Cone Disorders (INTROCONE)	Recruiting	Observational	Generating iPSCs from patients affected by cone disorders to assess the effect of intronic of unknown significance	NA

Abbreviations: CF, Cystic fibrosis; CFTR, Cystic fibrosis transmembrane receptor; DMD, Duchenne muscular dystrophy; iPSCs, induced pluripotent stem cells; IRD, Inherited retinal dystrophy; LUM/IVA, lumacaftor/ivacaftor; MS, Multiple sclerosis; ONB, Oligonucleotide blockers; RPE, retinal pigment epithelium; SMA, Spinal Muscular Atrophy.

Two clinical trials (EU Clinical Trials Register ID: 2015-001317-28 and 2014-000057-37) have used organoid-based measurements to assess the correlation between β2-agonist-induced CFTR function *in vitro* and long-term clinical treatment effect, including lung function and airway resistance. In addition, the potential systemic effect of the drug was assessed by incubating organoids with patient blood samples and measuring CFTR activity in the organoids. Another clinical trial (ClinicalTrials.gov ID: NCT03506061) obtained skin biopsy material and/or a blood sample from each subject and differentiated iPSCs were used to generate airway epithelial monolayers. This system was then used to test for candidate drug response. The ongoing clinical trial will compare responsiveness of iPSCs with any *in vivo* benefit, using clinically relevant endpoints such as forced expiratory volume (FEV1), in order to establish a robust correlation between *in vitro* and *in vivo* readouts. This trial also aims to recruit patients with rare/ultra-rare CF mutations and develop a new tool using iPSCs to identify patient populations most suitable for CF modulator therapy.

A clinical trial for Multiple Sclerosis (ClinicalTrials.gov ID: NCT06551649) that has not yet started recruiting, aims to develop a three-dimensional *in vitro* model of the neurodegenerative disease. Patient-specific and control organoids will be cultured with the secretome from human amniotic mesenchymal stromal cells (hAMSC) to assess the effects of this on inflammation, neurodegeneration and cell death. As mentioned, this trial is not yet recruiting, but it offers an exciting perspective for developing organoid models as a screening platform for novel therapeutics.

These studies highlight clinical trials that use CIVMs, such as iPSCs and organoids, to develop human cellular models for testing the efficacy and dosage of new drug candidates, establishing correlation between *in vitro* data and clinical outcomes, and to test the efficiency of cell/gene therapy approaches. In addition, these systems are also being used to build three dimensional cellular models of complex diseases, such as neurodegenerative diseases (e.g., MS), where the quantifiable cellular endpoints, such as neurite length, apoptosis, damage in myelin, number of inflamed microglial cells could be used as possible surrogate markers for clinical endpoints.

Apart from 11 interventional trials, there have also been nine observational trials employing CIVMs. These trials aim to establish patient-derived iPSC lines for creating personalised disease models for drug testing and understand disease mechanisms. For example, one trial (ClinicalTrials.gov ID: NCT03754088) aimed to generate iPSC lines from 3 people with CF and from 3 healthy subjects and validate an *in vitro* model of CF created using differentiated bronchial epithelium (from patient-derived iPSC lines). While the trial was completed in 2019, no study results have been posted on the registry. Similarly, four trials (NCT03853252; NCT01432847; NCT02162953; NCT04658251) have been undertaken to establish patient-derived iPSC models for retinal dystrophy disorders and to create models for testing the efficiency of gene therapy approaches for different diseases. One of these trials is interventional (NCT03853252) and the others are observational, however none have any results posted although three of these are still recruiting and only one (NCT02162953) is complete.

Out of the 18 clinical trials performed till now for the diseases that form the focus of this review (Table 1), seven have been completed, one was withdrawn, and another one was prematurely terminated. No specific reasons or information have been provided for the closure of the latter two trials. Additionally, for those trials that are completed, results are not publicly available in the clinical trials registry, despite encouragement for publication of these and a commitment to enhanced data sharing from the industry (Modi et al., 2023).

## 4 The way forward: using policy and regulation to drive human-based rare disease research and drug development

Globally, countries have designed laws, regulations, and programs to fast track the diagnosis and treatment of rare diseases (Fermaglich and Miller, 2023) (Regulation - 141/2000 - EN - EUR-Lex, 2024). However, rare diseases still represent a largely unmet need, given the number of rare diseases that have no, or very limited, therapeutic options. The adoption of patient-derived CIVMs for rare disease research holds transformative potential for drug discovery and patient stratification. To accelerate their use, we propose the following key recommendations (summarised in Table 2):

**TABLE 2:** Actionable recommendations to support human-based rare disease research and drug development.

Recommendation	Actionable stakeholders	Potential beneficiaries
1. Enhance investment in human biology-based basic research	Method developers, Funding agencies, Researchers	Patients, Researchers, Pharmaceutical companies
2. Increase data transparency	Drug sponsors, Clinicians, Researchers, Regulators	Regulators, Patients, Pharmaceutical companies, Researchers, Clinicians
3. Prioritise adoption of CIVMs	Regulators	Pharmaceutical companies, Patients
4. Create dedicated funding programmes for CIVMs	Funding agencies	Researchers, Clinicians, Method developers, Patients
5. Establish biobanks	Researchers, Patients, Clinicians	Researchers, Patients, Pharmaceutical companies, Method developers
6. Harmonise global policies	Regulators	Pharmaceutical companies, Method developers
7. Accelerate regulatory flexibility	Regulators, Pharmaceutical companies	Pharmaceutical companies, Method developers, Patients
8. Establish ‘Centres of Excellence’	Funding agencies, Researchers, Clinicians	Researchers, Clinicians, Patients
9. Accelerate indigenisation of innovation	Funding agencies, Method developers in Academia and Companies	Researchers, Pharmaceutical companies, Patients
10. Engage the public and involve patient associations	Researchers, Clinicians, Pharmaceutical companies, method developers	Patients, Patient associations, General public

The table lists some of the key actions that will be necessary to foster more investment in, and greater development and use of CIVMs for rare disease research and indicates responsible parties, in terms of stakeholders and beneficiaries.

### 4.1 Enhance investment in basic research

Sustained funding is essential to address the limited understanding of rare diseases’ mechanisms and potential treatments. While patient charities play a significant role (Group and Bainbridge, 2023), governments must provide consistent investment to ensure progress in diagnostics, registries, and personalized platforms created by CIVM developers.

### 4.2 Increase data transparency

Increased openness in clinical trial data, including reasons for withdrawn or terminated trials and both positive and negative results, can build confidence in CIVMs, reduce redundancy, and clarify their regulatory applications.

### 4.3 Prioritise adoption of CIVMs

CIVMs can significantly shorten time-to-market for rare disease treatments by demonstrating functional impacts (e.g., improved muscle contraction or reduced demyelination). Wider adoption will require cost considerations, demonstrable successes, and increased pharmaceutical sector integration (Alver et al., 2024).

### 4.4 Create dedicated funding programmes for development of CIVMs

Initiatives like the Horizon Europe’s ERDERA Partnership, the Horizon Europe call “Development of new effective therapies for rare diseases”, and the FDA’s Support for clinical Trials Advancing Rare disease Therapeutics (START) Pilot Program exemplify the importance of targeted funding to advance CIVM-based approaches, leveraging also clinical trial data and other technologies for efficient and patient-focused drug development.

### 4.5 Establish biobanks

Repositories of patient-derived materials, supported by ethical guidelines, can enable “*in vitro*” clinical trials, ensuring CIVMs are accessible and widely useable despite the scarcity of rare disease patients.

### 4.6 Harmonise global policies

Global adoption of CIVMs requires alignment of national and international guidelines, including ethical biobank standards and regulatory frameworks like those of the International Council for Harmonisation (ICH), to ensure global harmonisation in the production of safe and effective medicines and their effective delivery to patients.

### 4.7 Accelerate regulatory flexibility

Success stories, such as the FDA’s expanded approval of the CF drug ivacaftor based on *in vitro* data, highlight the need for adaptable regulatory pathways to integrate CIVMs into drug development processes (Kingwell, 2017).

### 4.8 Establish centres of excellence

Dedicated hubs, as seen in India’s rare disease centers, can drive innovation through multidisciplinary collaboration, education, and patient involvement, ensuring research aligns with real-world needs.

### 4.9 Accelerate indigenisation of innovation

Reducing reliance on global supply chains and building local capacity for CIVM development in middle- and low-income countries can ensure accessibility and foster sustainable, region-specific research ecosystems and unmet public health needs.

### 4.10 Engage the public and involve patient associations

Raising awareness of CIVMs’ potential and involving patient associations in research ensures alignment with patient needs, fosters trust, and enhances the impact of research outcomes.

By implementing these measures, CIVMs can become a cornerstone for advancing human-relevant approaches in rare disease research, addressing unmet medical needs and accelerating therapeutic discoveries.

## 5 Conclusion

The treatment of rare diseases presents a very challenging space, with each rare disease patient possessing one or more of thousands of rare and ultra rare mutations that can manifest as complex clinical symptoms. It requires a personalised and precise strategy of preclinical assessment that is not cost-intensive, as the current paradigm is also cost-prohibitive to most patients. CIVMs offer a powerful lens into human-specific drug responses, serving as a precise tool for advancing rare disease therapeutics, as outlined in this paper. Dedicated funding programs and a roadmap tailored to country-specific challenges will be crucial for advancing the development of technology and capacity in this area.

Tackling rare diseases using traditional (animal-based) research approaches is an inefficient, costly and resource-intensive strategy, and not likely to improve with additional investment. Given the urgent need for broader advancements in drug development processes, the adoption of human cell-based CIVMs presents an opportunity to greatly advance and refine these procedures at scale. By embracing these recommendations, we can accelerate drug development across all rare diseases, enabling more efficient, human-relevant therapeutics for the entire rare disease community.
